# Selective spinal anesthesia with hyperbaric prilocaine provides better perioperative pain control than local anesthesia for ambulatory inguinal hernia repair without affecting discharging time: a randomized controlled trial

**DOI:** 10.1186/s44158-022-00034-x

**Published:** 2022-01-31

**Authors:** Fabio Costa, Giuseppe Pascarella, Paolo Luffarelli, Alessandro Strumia, Gaspare Biondo, Chiara Piliego, Rossana Alloni, Felice E. Agrò

**Affiliations:** 1 Unit of Anaesthesia, Intensive Care and Pain Management, Department of Medicine, Campus Bio-Medico University of Rome, via Álvaro del Portillo 21, 00128 Rome, Italy; 2 Department of Pelvic Floor Surgery and Proctology, Campus Bio-Medico University of Rome, via Álvaro del Portillo 21, 00128 Rome, Italy; 3 Department of Specialistic General Surgery, Campus Bio-Medico of Rome, via Álvaro del Portillo 21, 00128 Rome, Italy

**Keywords:** Inguinal hernia repair, Spinal anesthesia, Ambulatory surgery, Hyperbaric prilocaine, Regional anesthesia

## Abstract

**Purpose:**

Local anesthesia is the most used anesthetic technique for inguinal hernia repair, despite its unpredictability. Selective spinal anesthesia with a short-term local anesthetic guarantees rapid recovery, predictable duration and low incidence of side effects. We tried to assess the efficacy of this neuraxial technique in ambulatory setting.

**Methods:**

One hundred thirty-two ASA I–III, aged > 18 patients scheduled for inguinal hernia repair have been randomized into two groups receiving unilateral spinal anesthesia with 40 mg of hyperbaric prilocaine (group A) or local anesthesia with mepivacaine (group B). Primary endpoint: intraoperative and post-operative NRS. Other outcomes: sensory block onset, need for opiates and deep sedation, surgery duration, and time to discharge.

**Results:**

Group A: intraoperative NRS was 0 in 100% of patients; post-operative maximum NRS was > 3 in 12.12% of patients. Group B: mean intraoperative NRS was 4; mean post-operative NRS was 2.5. Spinal anesthesia resulted superior in controlling both intraoperative and post-operative pain (*p* < 0.00001; *p* = 0.008). Mean time of the motor block resolution in group A was 98 ± 2 min. Mean time to discharge was not significantly different between groups. Surgical time was significantly different between the two groups (mean time of 37 ± 3.2 min group A; 54 ± 6 min group B—*p* < 0.00001).

**Conclusion:**

Spinal anesthesia group patients had significantly less pain than local anesthesia group, both intraoperatively and post-operatively, without differences in time to discharge, incidence of complications and with improvement of surgical time. More randomized controlled trials are needed to confirm this hypothesis.

**Trial registration:**

NCT05136534. Registered November 29, 2021—Retrospectively registered

## Introduction

Inguinal hernia repair involves more than 20 million patients annually all over the world, with an incidence of 10/100,000 in the UK and 28/100,000 in the USA, making it one the most common surgical procedures [1–3]. Moreover, approximately 10-15% of patients will have a recurrence and will require re-surgery [4], with a considerable impact on the social and health costs of this widespread disease.

Most of the inguinal hernia surgical procedures are performed on outpatients, as the modern concept of fast track surgery has led to decrease recovery time, surgical invasiveness, costs, recurrence rate, and post-operative pain [5].

The main options for inguinal hernia repair include open, laparoscopic, and robotic surgery. The open approach is the most chosen by the majority of surgeons, due to advantages in costs, ease of execution, hospital length of stay, and minimal invasiveness, making it perfectly suitable for ambulatory or day-surgery settings [6, 7].

The demand for anesthetic techniques that allow a fast patient discharge is one of the keystones of outpatients’ surgery. Therefore, local anesthesia is the most used anesthetic technique, despite its unpredictability and the potential patient’s discomfort [8].

Selective spinal anesthesia performed with a short-term hyperbaric local anesthetic could be a perfect solution, because it guarantees rapid sensory and motor block, predictable duration, and low incidence of side effects. It is usually well accepted by both patients and surgeons due to its high reliability, as it provides effective analgesia, with minimal side effects, rapid changeover times, and low costs [9].

Prilocaine is an amino-amide local anesthetic and it is characterized by intermediate potency with rapid onset time and short duration [10].

When a small dose of hyperbaric prilocaine is administered, with patient positioned in lateral decubitus, a selective unilateral block is induced in most of the patients [11].

This may result in faster patient discharge, making unilateral spinal anesthesia a good option for day-surgery procedures [12, 13].

Moreover, a selective spinal anesthesia is able to minimize the extent of the sympathetic block and reduce the incidence of hemodynamic impact, as demonstrated also in high risk patients undergoing unilateral surgery [14].

We conducted a randomized controlled trial study to assess the efficacy of selective spinal anesthesia with hyperbaric prilocaine compared to local anesthesia for ambulatory inguinal hernia repair.

Primary outcomes included intraoperative and post-operative pain scores. Secondary outcomes included onset of sensory block, need for intraoperative opioid and sedation, surgery duration, motor block duration, and time to discharge. Occurrence of adverse events was also recorded.

## Methods

### Enrollment

This study was performed between January 2019 and February 2020 at the Day Surgery Department of the Campus Bio-Medico University of Rome. This study was performed in line with the principles of the Declaration of Helsinki. Hospital ethics committee approval was obtained before starting patients’ enrollment (14.16 TS. ComEt CBM). The trial has been registered on ClinicalTrials.gov platform with identifier NCT05136534. This manuscript adheres to the applicable CONSORT guidelines.

**Fig. 1:**
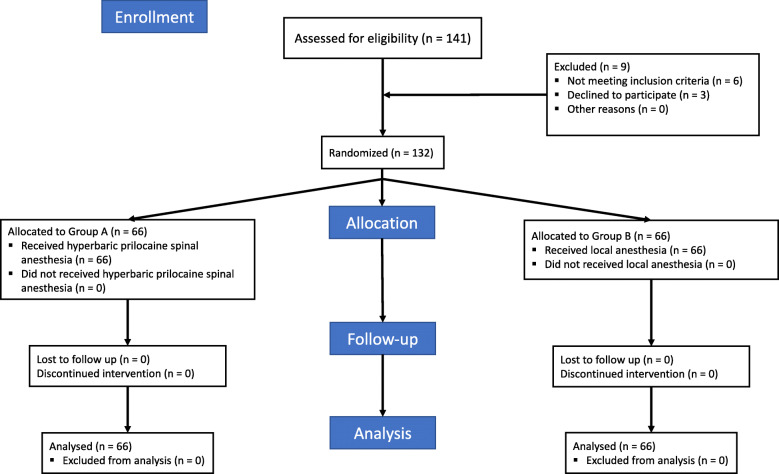
Consort 2010 flowchart

One hundred thirty-two ASA scale I–III, aged > 18 patients scheduled for inguinal hernia repair have been randomized into two groups receiving unilateral spinal anesthesia (group A) or local anesthesia (group B) (Fig. 1).

Randomization was achieved using computer-generated lists in blocks of 8 with a 1:1 ratio and treatment allocation was concealed using consecutively numbered, sealed, opaque envelopes. Patients with neurological disorders, allergy to local anesthetics, liver disease, serious cardiac conduction problems, severe anemia, cardiogenic or hypovolemic shock, congenital or acquired methemoglobinemia, primitive changes in coagulation, patients treated with class III antiarrhythmics (amiodarone), patients who did not suspended anticoagulants/antiplatelet agents and pregnant patients were excluded from this study.

All patients underwent inguinal hernia repair with the Trabucco’s technique [15], performed by the same surgical team. Every patient was adequately informed of the procedural sequence of anesthesia and surgery and signed informed consent before being enrolled in the study.

For both groups, patients received mild sedation with Midazolam 0.03 mg/kg i.v.; Paracetamol 1 gr and Ketorolac 30 mg i.v. were given before surgery as multimodal pre-emptive analgesia.

### Group A

Subarachnoid anesthesia was performed with a 27-G Whitacre needle at L1–L2 or L2–L3 interspace, with patients on the lateral decubitus corresponding to the side of surgery and in slight (5–10°) Trendelemburg position. After local anesthetic infiltration of the skin at the puncture site (2% lidocaine, 0.5–2 ml), the introducer was inserted in the middle point of the space between two spinous processes. The spinal needle was passed through the introducer and advanced till the subarachnoid space was reached, as confirmed by cerebrospinal fluid outflow.

Subsequently, 40 mg of 2% hyperbaric prilocaine were administered in the subarachnoid space, with a low-flow injection technique and the needle bevel turned laterally towards the sloping surgical side. Lateral decubitus together with the slight Trendelemburg position was maintained for 10 min [16].

Sensory and motor block were assessed respectively by ice-test (every minute) and numerical 0–3 Bromage Scale (15 min from spinal anesthesia execution and before surgery started). Target sensory block was any level higher than T10. A T10 level not reached would be considered a failed block.

### Group B

Patients underwent surgery with local anesthesia with mepivacaine 2–200 mg (10 ml) as initial dose—this was performed by the surgeon before skin incision and sufficient anesthesia of the surgical area was tested with the pinprick test. Further infiltration of 2 ml each time of local anesthetic was ensured in case of pain during the surgery, up to a maximum of 400 mg (20 ml) of mepivacaine.

In case of uncontrolled pain, fentanyl 50 mcg i.v. was given for a maximum of two intraoperative administrations. If pain persisted, a deep sedation was performed with a propofol continuous i.v. infusion.

### Outcome measurements

- Sensory block onset: from the end of spinal injection to the loss of cold sensation in the T10 dermatome.

Pain assessment was performed using a 0–10 numerical rating scale (NRS).

- Intraoperative NRS: was assessed every 10 min and every time the patients felt a pain intensity higher than 3 or asked for i.v. analgesia or sedation.
- Need for fentanyl or deep sedation: were recorded intraoperatively.
- Surgery duration: from the time-out check list to the last staple on the skin wound.
- Post-operative NRS: was assessed in post-anesthesia care unit (PACU) 60 min after the end of the operation.
- Motor block duration: was counted from spinal puncture to Bromage score “0”; was recorded in PACU every 10 min from the admission.
- Time to discharge: time from the end of surgery to the discharge from hospital (ability to walk and to oral intake, absence of complications, and spontaneous voiding were required).

The following complications were also evaluated: hypotension/hypertension (decrease/increase in systolic blood pressure by 30% compared to the baseline value), bradycardia (decrease in heart rate below 45 bpm), urinary retention, temporary urinary incontinence and transient neurological symptoms (TNS), post-operative nausea and vomiting, headache, and failure to discharge.

### Post-discharge protocol

Patients were discharged at home with the following drug regimen to control post-operative pain: acetaminophen 1 g every 8 h, ketorolac 30 mg per os on demand (90 mg/die maximum dose, in case of breakthrough pain with NRS > 5.

### Statistical analysis

The values of categorical variables are expressed as number and percentage. The parametric distributions of continuous variables are expressed in mean ± standard deviation (SD) and evaluated using the Kolmorogov-Smirnov test. The primary endpoint was compared between the two groups using the Mann-Whitney *U* test.

Secondary endpoints were tested using Student’s *t* test or Mann-Whitney *U* test, when appropriate. Nominal variables were compared using the Pearson chi-square test. The statistical significance level has been set for *p* < 0.05 values. All statistical analyses were carried out using R Statistical Software (https://www.r-project.org/).

## Results

A total of 132 subjects were included in the final analysis, randomly divided in 66 for each group.

Patients’ characteristics were similar between the groups and are resumed in Table 1, while post-randomization endpoints are resumed in Table 2.

**Table 1:** Patients’ characteristics

	Group A (n. 66)	Group B (n. 66)
Sex (M/F)	65/1	64/2
Age (years)	58 ± 8	57 ± 9
Body mass index (kg/m^2^)	27.2 ± 4.2	28.1 ± 6.1
ASA (most frequent value)	2	2
Hernia dimension (cm)	8.2 ± 4.2	8.4 ± 5.1

Values are reported as number (percentage) of subjects or mean ± standard deviation (SD)

*Group A* selective spinal anesthesia, *Group B* local anesthesia

**Table 2:** Post-randomization outcomes

	Group A (n. 66)	Group B (n. 66)	*p* value
Intraoperative NRS	0	4 (0–7)	< 0.0001
Post-operative NRS (60 min after surgery)	0 (0–2.75)	2.5 (0–4.75)	0.008
Sensory block onset (min)	3 ± 1.2	n/a	n/a
Motor block duration (min)	98 ± 2	n/a	n/a
Need for opioid administration	0	50 (%)	< 0.0001
Conversion to deep sedation	0	44 (%)	< 0.0001
Surgical time (min)	37 ± 3,2	54 ± 6.4	< 0.0001
Discharge time (min)	74 ± 5	75 ± 4.1	0,5625

Values are reported as number (percentage) of subjects, mean ± standard deviation (SD) or median and interquartile range (IQR)

*Group A* selective spinal anesthesia, *Group B* local anesthesia, *NRS* numeric rating scale

### Primary endpoints

#### Intraoperative and post-operative NRS

Intraoperative NRS was 0 in 100% of group A patients, while the post-operative maximum NRS was > 3 in only 8 patients (12.12%).

In Group B the mean intraoperative NRS pain score was 4 (moderate pain), but 29 patients experienced severe pain (NRS > 6). Mean post-operative NRS score (at 60 min from the end of the operation) was 2.5, but 13 patients experienced a post-operative NRS pain score > 6 which required additional pain killers (Fentanyl 50 mcg). Zero patients in the spinal anesthesia group needed additional opiates at 60 min.

Comparing the intraoperative and post-operative NRS pain score in the two groups, spinal anesthesia resulted significantly superior to local anesthesia in both cases, (*p* < 0.00001 and *p* = 0.008, respectively).

**Fig. 2:**
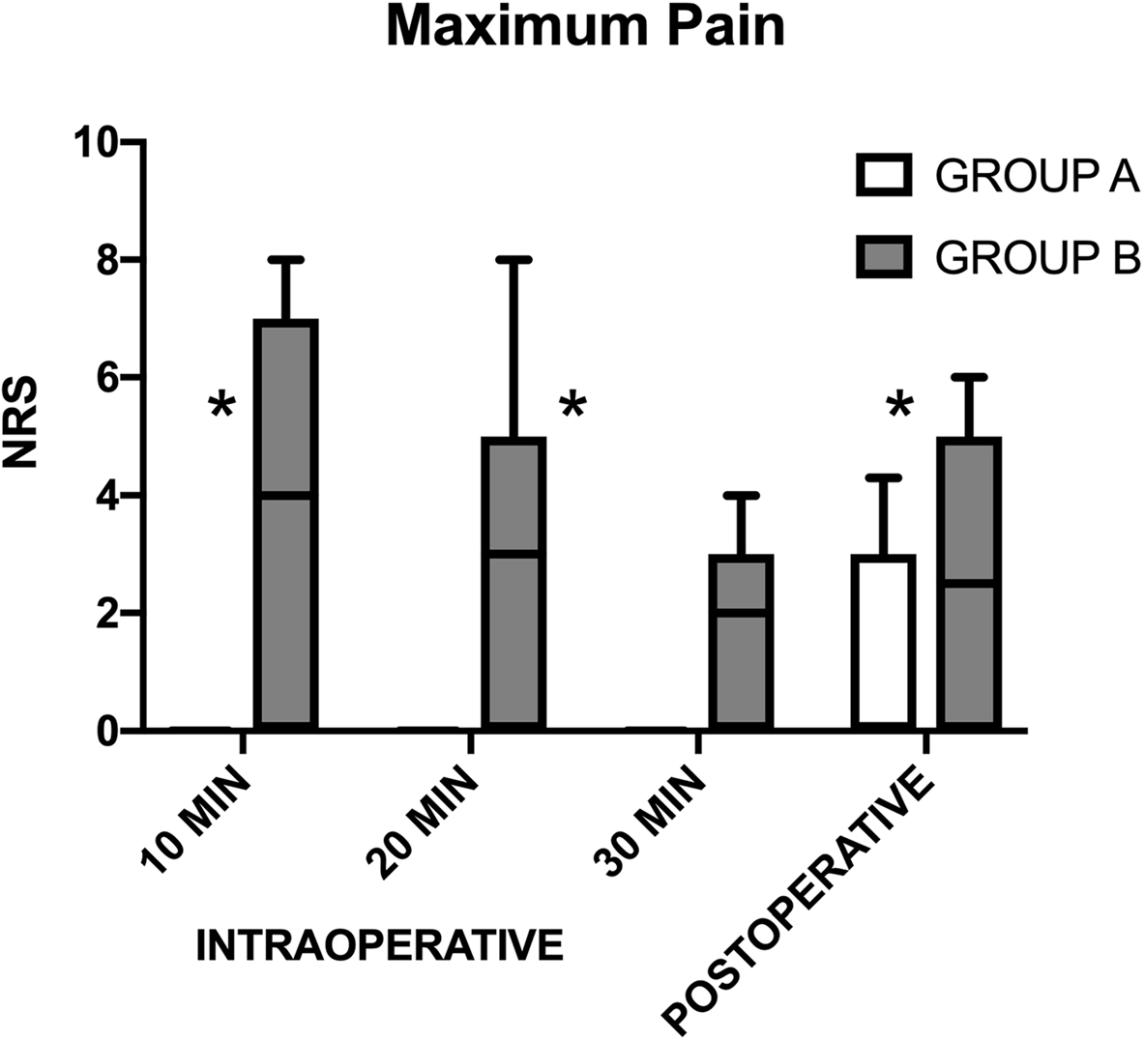
Perioperative Pain. The box plot shows pain scores during and after surgery performed by unilateral spinal anesthesia with hyperbaric prilocaine (Group A) vs. local anesthesia performed by surgeon (Group B). Data include maximum pain reported in a 0-10 Numeric Rating Sale, measured both intra and postoperatively. Postoperative values were recorded in PACU at 60 minutes after the end of surgery. Values are expressed as mean (horizontal bars) with 25th–75th (box) and 10th–90th (whiskers); *denotes statistical significance (*p* < 0.05); NRS: Numeric Rating Scale

Perioperative pain scores for the two groups are shown in Fig. 2.

### Secondary endpoints

#### Sensory block onset

Analyzing the efficacy of spinal block, time to obtain a unilateral sensory block to the T10 dermatome was 3 ± 1.2 min. The highest level of the sensory loss, 15 min after the spinal injection was T6 in 49% of patients (32), T7 level in 35% (23), T5 in 12% (8), T9 in 3% (2). One patient (0.15%) had highest sensory loss at T3 level.

#### Motor block duration

The mean time of the motor block resolution in group A (Bromage scale 0) was 98 ± 2 min from the execution of the spinal anesthesia.

#### Need for fentanyl or deep sedation

In group A no patients needed opioids or deep sedation during the surgery, while group B showed a rate of 50% of fentanyl administration and 44% of conversion to deep sedation.

#### Time to discharge

The reported mean time to discharge was not significantly different between Groups A and B, respectively 74 ± 5 and 75 ± 4.1 min (*p* = 0.5625) from the end of the operation.

#### Surgery duration

The surgical time was significantly different between the two groups, with a mean time of 37 ± 3.2 min in group A and 54 ± 6 min in group B (*p* < 0.00001).

No complications such as hypotension, nausea and vomiting, headache, transient neurological symptoms (TNS), transitory urinary incontinence, or urinary retention were reported in both groups; there was only one case of bradycardia in the group A, promptly regressed with the administration of 1 mg of Atropine.

## Discussion

Inguinal hernia repair is one of the most performed surgical procedure worldwide [17]. Innovations in surgical and anesthetic techniques have allowed to significantly decrease the impact on the patient, permitting to perform the procedures in an ambulatory setting [5]. Local anesthesia seems to be the most recommended anesthetic choice in term of cost-effectiveness [18], but some problems still remain: it is proved to be effective for small and reducible hernias, and depends on surgeon experience [19]. Sometimes could be insufficient and intraoperative sedation may be required [20].

Patients’ comfort and pain control would be guaranteed with spinal anesthesia, even if it is affected by potential complications, such as hypotension, post-dural puncture headache (PDPH), urinary retention, and, the most feared in the ambulatory setting, prolonged lower limbs paralysis. Those negative features are commonly related to long acting local anesthetic agents, such as bupivacaine or ropivacaine. Nevertheless, short acting agents such as lidocaine and mepivacaine are not recommended because of their high correlation with transient neurological symptoms (TNS).

Hyperbaric prilocaine is a short acting drug, not associated with TNS, that allows selective unilateral spinal anesthesia with lower incidence of complications.

With this study, we compared the use of selective, unilateral spinal anesthesia with hyperbaric prilocaine, with local anesthesia with mepivacaine performed by the surgeon, in 132 patients who underwent open inguinal hernia surgical repair.

Our data showed how in spinal anesthesia group (group A), patients had significantly less pain than local anesthesia group (group B), both intraoperatively and post-operatively, enhancing patients’ comfort and surgical experience. Group A patients required neither intraoperative opioids nor sedation. On the contrary, almost half of the group B patients required intraoperative opioids and over 40% of them had to be deeply sedated. Similar results arises in another study from Palumbo et al [21]. On the other hand, data from a review by Prakash et al. [22], including 10 RCTs and 1379 patients, are strongly in support of local anesthesia, showing higher intraoperative pain, higher failure rate as well as higher urinary retention rate in spinal groups. Other studies revealed a higher rate of similar complications with spinal anesthesia [22–24], but those results usually match with low, bilateral, neuraxial blocks and use of long acting agents, and none of the studies included were carried out with hyperbaric prilocaine. In our study, there was no complications described, and we reported only one case of bradycardia rapidly regressed after atropine administration.

Low dosage prilocaine use, combined with the right puncture level, the kind and size of the needle, the injection technique, and the experience of the anesthetist, surely contributed to reach these results. A wrong injection technique might be sufficient to affect the block effectiveness. We performed all the injections very slowly with the bevel of a 27-G Whitacre point needle oriented downward in order to obtain a complete lateralization of the block [16]. Furthermore, we administered the agent with the patients in Trendelemburg position, avoiding drug pooling in the lower dural sac (potential cause of block failure and urinary retention) and enhancing its cranial distribution to the thoracic roots, prolonging abdominal analgesia. We found neither cases of urinary retention, nor other complications that could prolong time to discharge (groups A and B, respectively 74 ± 5 and 75 ± 4.1min *p* = 0.5625). Ultimately, the advantageous pharmacokinetic of the prilocaine made the rapid recovery possible. The rapid recovery we obtained in our study is consistent with the data published in a review by Boublik et al. [9], who analyzed the use of prilocaine for ambulatory surgery in 5486 cases. Dosage between 30 and 60 mg appears to be the safest in terms of unilateral anesthetic success and time to recovery [9, 25, 26]. Differently, in several other studies which compared local anesthesia with spinal anesthesia performed with other drugs [23, 24, 27], recovery time and subsequently time to discharge has been found significantly longer in spinal groups.

In our research, time to discharge is measured from the end of the surgery to the exit from the hospital. Fundamental criteria for readiness to discharge included voiding and ambulation. Most of the group A patients were ready to ambulate and were discharged home approximately 30 min after the end of surgery. A large proportion of the group B patients needed to recover from a deep sedation, prolonging the time to discharge.

Regarding the surgery time, in their review, Prakash et al. found no difference between the groups.

Moreover, most of the available literature shows no differences in surgical time [8, 22, 27] or even a shorter surgical time for the local anesthesia patients [23, 24]. The contrast of this results with our finding is probably related to the extreme variability in local anesthesia methodology between different authors, either for technique and time required or for its efficacy and need for further intraoperative infiltrations and time wasting. In our research, local anesthesia technique was not standardized. As a result, effectiveness of local anesthesia might have been influenced by operator experience and personal methodology and, being local anesthesia time included in surgery time, surgery time could have changed accordingly. Conversely, spinal anesthesia time is not part of the surgery time, being the neuraxial block performed before the surgery, outside the operating room. This is a major bias of our study. Differences in local anesthesia mixtures and patients’ variability in pain tolerance, may also explain these discrepancies between literature’s data and ours.

Beyond all these considerations, in our facility spinal anesthesia with prilocaine has proven to be more effective and efficient than local anesthesia. Considering our standard surgical session of 6 h (8 a.m.–2 p.m.) for 5 procedures scheduled, a time saving of 17 min per procedure, resulted in a total saving of about 90 min that perfectly fit 2 extra procedures, resulted in a great improvement of the workflow and of the efficiency of the operating room increasing cost-effectiveness of the procedure. It has to be said that, in differently organized units, where performing spinal anesthesia outside the operating room before the intervention is not feasible, including the time for spinal anesthesia would make the operative time longer, perhaps negating the benefit on surgical time for spinal anesthesia.

Another bias of our study is the fact that neither the operator nor the patient could be blinded. Additionally, we have evaluated post-operative pain just at 60 min after surgery, which is a quite short time to evaluate post-operative analgesia, especially considering that local anesthesia may give a longer lasting benefit than spinal anesthesia; a home readiness scoring every half-hour and a long-term observation of post-operative pain would have returned even more interesting data. Although our data suffers from these limitations, they encourage us to further investigate and better define whether unilateral spinal anesthesia with prilocaine should be placed or not beside local anesthesia in the open inguinal hernia management guidelines. More randomized controlled trials are needed to confirm this hypothesis.

## Conclusion

This prospective randomized study suggests that unilateral spinal anesthesia with hyperbaric prilocaine, when compared to local anesthesia for open inguinal hernia repair, may improve intraoperative and post-operative pain control, reducing surgical time and increasing patient’s comfort.

Moreover, the short discharging times and the irrelevant incidence of complications make this anesthetic procedure suitable for ambulatory inguinal hernia surgery. More randomized studies are needed to confirm our results.

## Data Availability

The datasets analyzed during the current study are available from the corresponding author on reasonable request.
